# Prevalence of Clinically and Empirically Defined Talents and Strengths in Autism

**DOI:** 10.1007/s10803-014-2296-2

**Published:** 2014-11-06

**Authors:** Andrée-Anne S. Meilleur, Patricia Jelenic, Laurent Mottron

**Affiliations:** The University of Montreal Center of Excellence for Pervasive Developmental Disorders (CETEDUM), Hôpital Rivière-des-Prairies, 7070 Perras Blvd., Montreal, QC H1E 1A4 Canada

**Keywords:** Perception, Savant, Talent, Block, Pitch, Expertise

## Abstract

Outstanding skills, including special isolated skills (SIS) and perceptual peaks (PP) are frequent features of autism. However, their reported prevalence varies between studies and their co-occurrence is unknown. We determined the prevalence of SIS in a large group of 254 autistic individuals and searched for PP in 46 of these autistic individuals and 46 intelligence and age-matched typically developing controls. The prevalence of SIS among autistic individuals was 62.5 % and that of PP was 58 % (13 % in controls). The prevalence of SIS increased with intelligence and age. The existence of an SIS in a particular modality was not associated with the presence of a PP in the same modality. This suggests that talents involve an experience-dependent component in addition to genetically defined alterations of perceptual encoding.

**Electronic supplementary material:**

The online version of this article (doi:10.1007/s10803-014-2296-2) contains supplementary material, which is available to authorized users.

## Introduction

The presence of a discrepancy between the apparent level of cognitive or adaptive functioning and at least one competence was reported in neurodevelopmental syndromes as early as two centuries ago (e.g., Gottfried Mind, 1768–1814). It was included in Kanner’s (1943) and Asperger’s (1944) seminal description of what is currently known as the autism spectrum. The variety of assumptions regarding the relation between these talents and intelligence is reflected in their multiple, overlapping labels: idiot savant, savant syndrome, splinter skills, islets of abilities, special isolated skill (SIS), peaks of abilities, uneven cognitive profile, and cognitive disharmony. Reports of autistic talents vary depending on the criteria used to define them. They can be characterized as an area of functioning in the average or superior range in an individual with intellectual disability. Alternatively, they may involve a discrepancy between performance level in a particular domain and an individual’s general level of cognitive functioning, or that expected for his/her age or developmental level. With few exceptions (Bennett and Heaton 2012; Bouvet et al. 2014a; Heaton et al. 1999), most case studies on talent are limited to the description of outstanding abilities noticed by the subject’s close relatives, such as precocious reading, or to domains of skills randomly revealed by cognitive tests, such as visuospatial abilities. Most studies may therefore underestimate talents that are either concealed or not examined in typical cognitive tests. Moreover, there is considerable variation in the reported prevalence of talents among studies, from the most quoted figure of 10 % of autistic individuals in Rimland’s historical study (Rimland 1978), to 71 % in another study by Rapin 1996. Due to these inconsistencies, it is unclear whether talents are a specific feature of the autistic phenotype. Nonetheless, autistic individuals make up at least 50 % of people presenting such abilities, suggesting that the development of discrepant skills is more likely to be favored in autism than in other neurodevelopmental conditions (Heaton and Wallace 2004; Miller 1999; O’Connor and Hermelin 1989). The variability in the reported prevalence of talents could be explained by differences in the characteristics of study participants (age, sex, intelligence, language level), and by differences both in the definition of talents and in tools used to measure performance level for particular abilities. Table 1 summarizes studies reporting the prevalence of talents in autistic individuals, including the initial reports of Kanner (1943) and Asperger (1944).

**Table 1:** Group studies investigating numerous talents and strengths in ASD, including Kanner and Asperger’s seminal descriptions

	N, diagnosis; mean age (range); mean intelligence level (range)	Skill definitions	Skill assessment tools; information sources	Skill types (prevalence in %)
Kanner (1943)	11 autistic individuals; 5.38 (2.33–11); From “severe intellectual defect” to “superior intelligence”	Observations ranging from “At the age of 1 year he could hum and sing many tunes accurately.” to “…and had an excellent rote memory.”	Direct observation; Clinician and parental reports	Any talent (54.5); Memory (54.5); Music (36.3)
Asperger (1944)	4 autistic individuals; 8.25 (6–11); IQ n/a	Observations ranging from “His extraordinary calculating ability had been reported by the parents and verified by us.” to “He was an excellent speller and never made mistakes.”	Direct observation; Clinician and parental report	Any Talent (75); Memory (50); Calculation (25); Spelling (25)
Rimland (1978)	540 autistic individuals; Age and IQ n/a	“…any “special abilities” the child may display.”	Postal survey; Parental report	Any talent (9.8)
Rapin (1996)	51 “High-functioning autistic individuals”; 4.8 (range n/a); NVIQ 102.9 (range n/a)	“…whether they (parents) felt that their child had unusually well developed abilities in memory, mathematics, recognition of letters and numbers, music or motor skills.”	Questionnaire; Parental report	Memory (70.6); Number and Dates (45.1); Puzzle/spatial skills (35.3); Music (27.5); Fine motor (21.6); Letters/numbers (25.5); Other (31.4)
	125 “Low-functioning autistic individuals”; 5.0 (range n/a); NVIQ 45.6 (range n/a)			Memory (44); Dates (13.6); Puzzle/spatial skills (26.4); Music (23.2); Fine motor (14.4); Letters/numbers (9.6); Other (17.6)
Bolte and Poustka (2004)	254 autistic probands; 15.54 (6–49); FSIQ 72.56 (32–129)	ADI-R Score of 3 or 4 coded on “current” behavior: performance level above the participant’s general level of cognitive functioning and above that expected for their age, with (4) or without (3) functional or adaptive use of skill in daily life	ADI-R special isolated skills items (106–111); Parental report	Any Talent in memory, music, computation, reading, visual-spatial and/or drawing (13)
Howlin et al. (2009)	*Assessment 1* 87/137 autistic individuals; 24.1 (11–48); FSIQ 77.52 (39–130)	Exceptional cognitive skill in any of the Wechsler subtests (1 SD ≥ population norms and 2 SD ≥ participant’s mean)	Wechsler subtests; Psychometric assessment	Strength in cognitive skill for at least one Wechsler subtest (26.4)
	*Assessment 2* 93/137 autistic individuals; 34.2 (21–55); PIQ 73.56 (29–135)	Outstanding skill/knowledge definitely above subject’s general level of ability and above that of age-matched individuals from the general population	Questionnaire; Parental report	Any Talent in memory, music, computation (including calendrical calculation) and/or visual-spatial (25.8)
	137/137 autistics n/a (11–55) PIQ 69.9 (28–135) VIQ 77.5 (7–134)	See definitions above	Questionnaire and Wechsler subtests	Any Talent and/or cognitive skills (28.5)
Jones et al. (2009)	100 ASD; 15.6 (14.8–16.9) FSIQ 84.3 (50–119)	Test performance highly discrepant from own general intellectual functioning	Wechsler Objective Reading and Numerical Dimensions and test of Word Reading Efficiency; Psychometric assessment	Strength in literacy and/or mathematics (72.7)
Bennett and Heaton (2012)	125 ASD; 10.0 (3–20); IQ n/a but 7.9 % had intellectual disability	One or more skills that were outstanding given their functioning skills based on nine structured and open-ended questions	Questionnaire; Parental report	Any Talent (42); Memory/knowledge (28); Mathematical/numerical (15.2); Artistic (9.6); Music (9.6); Reading/vocabulary (9.6); Spatial (8.8); ICT (8.8); Mechanical (3.2); Other (4.8)
Current study	*Study 1* 254 autistic individuals; 11.35 (2–39) FSIQ 87 (40–130) (n = 171)	ADI-R Score of 2 or 7 coded on “current” or “ever” behavior: Performance level above the participant’s general level of cognitive functioning and above that expected for their age, with (7) or without (2) functional or adaptive use of the skill in daily life.	ADI-R special isolated skills items (88–93); Parental report	Any Talent (62.6); Memory (52.5); Visuospatial (32); Reading (22.4); Drawing (17.5); Music (16.9); Computation (16.7)
	*Study 2* 43/254 autistic individuals; 20.81(14–36); FSIQ 105 (71–125)	Perceptual peak on experimental tasks (1 SD ≥ population mean)	Modified block design and Pitch Discrimination Tasks; Experimental testing	Strength in perceptual performance for at least one experimental task (57.5)
	Same 43 subjects	See definitions above	ADI-R special isolated skills items (88–93) and Experimental Tasks	Any Talent and/or perceptual peak (88.4)

*ASD* autism spectrum disorders, *FSIQ* full scale IQ, *VIQ* verbal IQ, *PIQ* performance IQ, *NVIQ* non-verbal IQ, *ADI-R* autism diagnostic interview-revised

Outstanding skills in autistic individuals may involve domain-general abilities found at the group level (generally referred to as peaks or islets of abilities) or domain-specific abilities at the individual level, often called savant syndrome. We will herein refer to domain-general abilities as “strengths” and domain-specific abilities as “talents” to avoid the discriminative assumptions behind these terms. Strengths are indicative of altered information processing, whereas talents are more influenced by practice and expertise (Mottron et al. 2013a). A cognitive strength is defined as a discrepancy between an individual’s capability in a non-verbal standardized task, mostly perceptual in nature (e.g., Wechsler’s block design subtest), and their average cognitive performance, which is measured with a combination of tasks that are frequently verbally mediated [e.g., Wechsler’s global IQ (GIQ)]. Although the term ‘strength’ refers to performance level in one particular type of task, it aims to reflect “domain general” strengths, which can be applicable to a large array of tasks. By contrast, a clinically defined talent, which is referred to in the ADI-R (Lord et al. 1994) as a “Special Isolated Skill” (SIS), is defined as a discrepancy between a domain of performance and overall adaptive or functioning level. Depending on the definition used, both strengths and talents can be inferior, equivalent or superior to the performance level of typically developing (TD) individuals of comparable age in the general population.

Although both strengths and talents are probably characteristic of autism, these two measures do not seem to overlap, and the relationship between them is poorly documented. Strengths are frequently considered to be implicated in the occurrence of talents, although the demonstration of a direct link between these two types of abilities is still lacking (Caron et al. 2006; Heaton et al. 2008b; Vital et al. 2009). Howlin et al. (2009) conducted the first study of the association between autistic strengths, defined with standardized cognitive tests, and talents, defined clinically with parental reports, within a single population. They investigated the nature and prevalence of strengths and talents in 137 autistic individuals at two different time points. These authors also examined whether the presence of outstanding skills varied according to sex and assessed the relationship between these skills and the presence of repetitive, restricted and stereotyped behaviors during development. They found that 25.8 % of autistic individuals had talents, 26.4 % had cognitive strengths, and 28.5 % had one or both types of skills. They reported only a modest overlap (8.6 %) between talents and strengths. Although this study represents a considerable advance in our understanding, it leaves important issues unanswered. First, it did not examine what is arguably the most common strength, a perceptual peak (PP) of performance in pitch discrimination. Also, the authors limited their search of talents to “current” presentations; therefore, talents closely related to development, such as early attainment of academic milestones in reading, were left undocumented. Here, we carried out two studies designed to investigate the nature and prevalence of talents (Study 1) and perceptual strengths (Study 2) in a large group of strictly defined autistic individuals across a wide range of age and intelligence levels. We investigated visual and auditory perceptual strengths in Study 2 with experimental tasks that included a modified block design (BD) task and a pitch discrimination task. Enhanced visual pattern detection and manipulation (Mottron et al. 2013b; Stevenson and Gernsbacher 2013) and superior pitch discrimination (Bonnel et al. 2003; Heaton et al. 2008b; O’Connor 2012) in autistic people, has been replicated in numerous studies. We sought to address the following questions: (a) What is the prevalence of outstanding abilities (Study 1: talents, Study 2: perceptual strengths)? (b) What are the predisposing factors (e.g., intelligence, age, sex) for each type of outstanding ability? and (c) What is the pattern of co-occurrence of outstanding abilities across modalities (visual and auditory)? Answers to these questions will improve our understanding of the contribution of expertise and educational opportunities versus innate predispositions to the development of outstanding skills. In keeping with the current consensus on language in autism research, the term “autistic individual” rather than “person with autism” is used in a respectful way (Pellicano and Stears 2011; Sinclair 1999).

## Methods

### Participants

Only autistic participants were included in Study 1. The research cohort was composed of 265 autistic individuals enrolled at the University of Montreal Centre of Excellence for Pervasive Developmental Disorders Research Database with available data for the “Special Isolated Skills” (SIS) section of the ADI-R. None of the participants satisfied the DSM-IV Asperger criteria. All autistic participants were diagnosed in the Specialized ASD Clinic at the Rivière-des-Prairies Hospital and consented to the use of their assessment data for research purposes. This clinic receives all suspected ASD individuals living in the eastern part of Montreal (Quebec), in addition to adults from the greater Montreal area. Trained clinicians diagnosed participants with standardized tools (ADI-R and/or ADOS) and clinical expert judgment based on DSM-IV criteria. Ten (3.8 %) participants with ASD associated with medical conditions (e.g., fragile-X syndrome) or other neurological conditions (e.g., epilepsy) were excluded, along with one other subject, who was considerably older (65 years old) than the rest of the group. In the end, talents, defined as SIS, were investigated in Study 1 in 254 autistic individuals (223 males and 31 females). Of these, 150 (59 %) had completed the Raven progressive matrices (RPM) (Raven and Summers 1986; Spreen and Strauss 1991) and 171 (67 %) had completed either the child (WISC) (Wechsler 1991, 2003) or adult (WAIS) (Wechsler 1997, 2008) version of the Wechsler’s Intelligence Scales. Overall, 138 (54 %) completed both tests (Table 2a).

**Table 2:** Descriptive Characteristics of participants for a. Study 1: Talents, i.e. “Special Isolated Skills” and b. Study 2: Strengths, i.e. “Perceptual Peaks”

	Talents (i.e. ADI-R “Special Isolated Skills”)			
	n	Mean	Range	SD
a.				
Age in years	254	11.35	2–39	8.00
Wechsler’s GIQ z-score	171	−0.85	−4.00 to 2.00	1.28
RPM z-score	150	0.54	−2.33 to 2.33	1.11
RPM raw score	150	41.2	7–60	11.99

	Strengths (i.e. “Perceptual Peaks”)						
	Autistics			TD Controls			
	(n = 43, 8 females: 35 males)			(n = 38, 5 females: 33 males)			
	Mean	Range	SD	Mean	Range	SD	*p*
b.							
Age in years	20.81	14–36	0.94	20.24	14–25	0.68	0.606
Wechsler’s GIQ z-score	−0.39	−1.93 to 1.73	0.99	0.48	−1.33 to 2.07	0.87	<.001**
RPM z-score	0.70	−1.30 to 2.30	0.85	0.42	−1.00 to 1.80	0.74	0.115
RPM raw score	46.33	27–60	7.00	45.71	31–57	7.09	0.696

*RPM* Raven progressive matrices (maximum raw score is 60), *GIQ* global IQ from Wechsler’s Intelligence Scales, *SD* standard deviation

** *p* < 0.001

In Study 2, PP of ability were assessed in both autistic and TD individuals. A sample of 46 autistic individuals was randomly selected from individuals in Study 1 who were over 14 years old with an RPM above the 25th percentile. These criteria regarding age and cognitive level (i.e., average range) were selected to ensure adequate understanding of the task instructions. Forty-six TD controls meeting the same inclusion criteria were also randomly selected from the CETEDUM Research Database. TD controls included in this database are recruited through public advertisements distributed to hospital employees, parents and friends of research staff as well as colleges and universities in the Montreal area. Neither TD participants nor their first-degree relatives had any history of ASD or other neurodevelopmental or neurological conditions. Autistic and TD control groups included in Study 2 were matched for RPM rather than the Wechsler’s scale to prevent the effect of circularity on peaks of ability that are defined relative to Wechsler’s GIQ. Participants with more than 5 years of formal musical and/or visual art education were removed from the analyses (three autistic individuals and eight controls), because training may influence perceptual performance (Micheyl et al. 2006; Tervaniemi et al. 2009). The final sample consisted of 43 autistic individuals and 38 TD controls (Table 2b). The onsite ethics committee approved the research and all subjects gave written consent to participate.

### Special Isolated Skills (SIS)

Special isolated skills (SIS) were determined with questions 88–93 of the ADI-R. SIS were conservatively defined as a coding of 2 or 7 (equivalent to 3 or 4 in earlier ADI versions), corresponding to a performance level above the participant’s general level of cognitive functioning and above that expected for their age, with (7) or without (2) functional or adaptive use of the skill in daily life. A SIS was recorded as present if at least one outstanding ability occurred in either time frame explored, namely “current” and/or “ever”. The prevalence of SIS in the “ever” and “current” time frames are available as supplementary material (Table S1).

### Perceptual Peaks (PP)

**Fig. 1:**
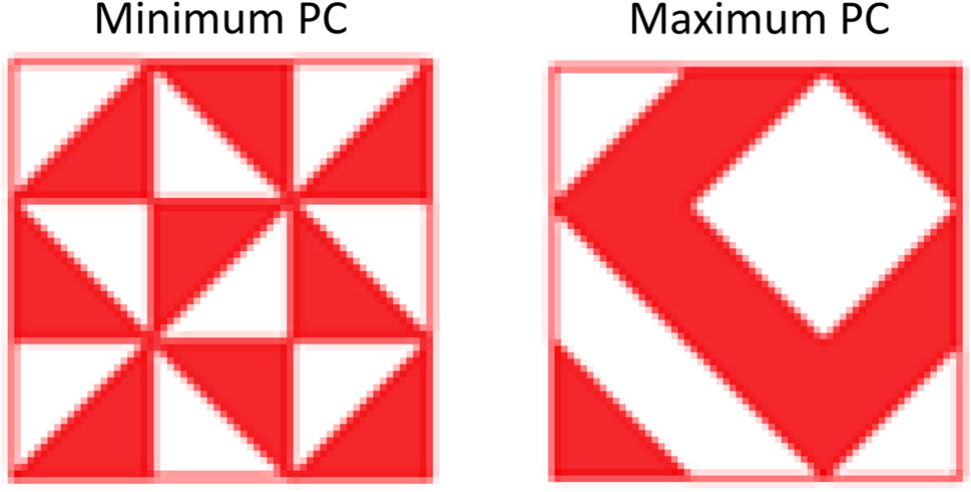
An example of two models of the modified block design task for the minimum (*left*) and maximum (*right*) perceptual coherence (PC) levels

Two perceptual tasks in which autistic individuals consistently perform better than TD controls were used to measure PP. This empirical study was part of a larger study on auditory and visual perception, containing a total of four different tasks (Meilleur et al. 2014). A pitch discrimination task (Bonnel et al. 2010) was used to measure the performance of low-level auditory perceptual processes. Frequency discrimination thresholds were measured at three levels (500, 1,000, 1,500 Hz), presented in a random order by a single adaptive staircase procedure and Harvey’s ML-PEST (Harvey 1997). Peak analyses were conducted on performance in the 500 Hz condition, because this condition was the most difficult condition and discriminated best between groups. Participants also completed a visual, modified BD task composed of three levels of perceptual cohesiveness (PC) (Caron et al. 2006). The PC level was manipulated by changing the number of opposite-colored edges, or *edge cues*. The higher the PC, the more difficult the task, and the larger the difference in performance between autistic and non-autistic individuals (Caron et al. 2006). The “Maximum PC” condition was used for peak analysis (Fig. 1), for similar reasons as the auditory task. In both tasks, participants who could not complete practice trials, obtain a minimum level of performance on initial test items, or complete enough trials to obtain a valid threshold measure, were excluded (Pitch task: three controls and seven autistic individuals; BD task: two controls and six autistic individuals). A PP was recorded as present if it occurred in one or both tasks.

Peak analysis was conducted with the modified *t* test program^1^ (Crawford et al. 2009; Crawford and Howell 1998). This statistical test was chosen to compare a single participant to a comparison group with a mean and standard deviation estimated on a small sample. For each experimental task, individual scores were entered into the program along with the mean, standard deviation and sample size of the control group. The program generated standardized levels of performance for each individual, expressed as a percentile (or ranked level of performance). Each participant’s performance ranking was then transformed into a z score and mapped onto a normal distribution. Similarly, each participant’s Wechsler’s GIQ score, expressed as a standard score, was transformed into a z score. A ‘relative’ performance level, indicative of PP, was calculated for each individual by subtracting the participant’s baseline level of cognitive functioning from his/her level of performance on the experimental perceptual task. A relative ‘peak’ was defined as a performance level at least 1 standard deviation above the individual’s general cognitive functioning level (GIQ). Each task was then coded with a binary score based on the absence (score = 0) or presence (score = 1) of a PP.

### Statistical Analyses

All analyses were conducted with SPSS Version 21 (2012). Preliminary analyses were conducted in Study 2 to examine between-group differences in standardized performance scores for each task and between-task differences in performance for each group. Analyses of prevalence and predisposing factors were then conducted for Study 1 and 2 separately, and analyses of co-occurrence of peaks were conducted only as part of Study 2. A critical *p* value of *p* = 0.01 (instead of *p* = 0.05) was used for all analyses to control for the multiplicity of tests and provide an adjustment that was not overly conservative. Cognitive results (standard scores for Wechsler’s IQ and percentiles for RPM) were converted to z scores assuming a normal distribution.

### Prevalence

For each study, the prevalence of SIS and PP was calculated as the percentage of peaks observed within the entire sample of autistic individuals and/or TD controls. Groups with and without peaks were compared with independent *t* tests and Chi square tests. Logistic regression analysis including group as a factor was conducted to determine the likelihood of an individual having a strength (SIS or PP) based on their group (autistic vs. TD control). The McNemar test was used to compare the prevalence of different strengths within a group.

### Predisposing Factors

Logistic regression analysis was then conducted to determine the likelihood of an individual having a strength (SIS or PP) based on various factors including age, intelligence (Wechsler’s GIQ or RPM), sex, and group (autistic and TD control). Dependent variables included binomial coding (presence or absence of SIS and PP).

### Co-occurrence of Outstanding Abilities in Autistic Individuals

The pitch discrimination peak was included in the logistic regression model as the independent variable, and the BD completion peak was included as the dependent variable to determine the likelihood of having a second PP when one was already present. Similar analyses were conducted to examine the co-occurrence of particular strengths within the same modality. These analyses explored whether a history of a clinically defined visuospatial or drawing SIS was associated with a high chance of having an empirically defined peak in the BD task and whether a history of SIS in music was associated with a high chance of having a PP in the pitch discrimination task.

## Results

### Study 1: Special Isolated Skills (SIS)

#### Prevalence

**Fig. 2:**
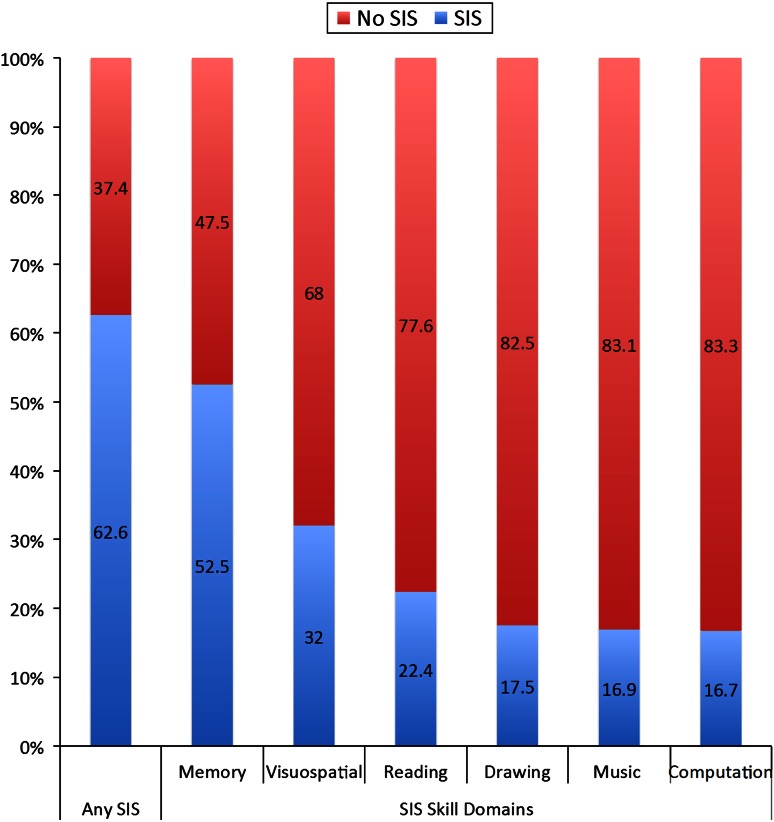
Graph showing the percentage of the sample from Study 1 with (*blue*) and without (*red*) reported talents, or “Special Isolated Skills” (SIS), in any domain and in each of the six ADI-R SIS domains separately

We examined the prevalence of SIS in 254 autistic individuals. Table 3a shows the characteristics of the participants including age and intelligence level and Fig. 2 illustrates the prevalence by domain. The proportion of autistic individuals with at least one reported SIS was 62.6 % (159/254). Special memory skills were the most frequently reported SIS, and were found in 52.5 % (127/242) of autistic individuals. Among autistic individuals with a clinically defined special skill, 71.7 % (114/159) had more than one SIS. We also examined how frequently SIS are lost, which showed that SIS are lost in 5–22 % (mean of 12 %) of individuals, depending on the type of skill. However, the loss of all abilities was uncommon (5 %) (Table S2).

**Table 3:** Descriptive characteristics of participants with or without outstanding skills and between-group statistics for a. Special isolated skills (SIS) and b. Perceptual peaks (PP)

	Special isolated skills (SIS)		Statistics	*p*
	With	Without		
a.				
Autistic individuals				
N (%)	159 (62.6)	95 (37.4)	Binomial test	<0.001**
Male (%)	141 (63.2)	82 (36.8)	χ^2^(1, N = 254) = 0.310	0.578
Wechsler’s GIQ z-score (SD)	−0.67 (1.28)	−1.36 (1.13)	t(169) = −3.208	0.002*
RPM z-score (SD)	0.68 (1.08)	0.04 (1.07)	t(146) = −3.112	0.002*
Age in years (SD)	13.36 (8.25)	7.97 (6.28)	t(237.455) = −5.878	<0.001**

	Perceptual peaks (PP)		Statistics	*p*
	With	Without		
b.				
Autistic individuals				
N (%)	23 (57.5)	17 (42.5)	Binomial test	0.430
Male (%)	20 (87)	13 (76.5)	Fischer Exact	0.432
Wechsler’s GIQ z-score (SD)	−0.73 (0.77)	0.25 (0.94)	t(38) = 3.620	0.001*
RPM z-score (SD)	0.85 (0.63)	0.70 (1.04)	t(38) = −0.568	0.574
Age in years (SD)	20.00 (5.48)	21.47 (6.30)	t(38) = 0.787	0.436
TD controls				
N (%)	5 (13.2)	33 (86.8)	Binomial test	<0.001**
Male (%)	5 (100)	28 (84.8)	Fischer Exact	1.000^†^
Wechsler’s GIQ z-score (SD)	0.19 (1.23)	0.53 (0.82)	t(36) = 0.815	0.421
RPM z-score (SD)	0.34 (0.70)	0.43 (0.76)	t(36) = 0.258	0.798
Age in years (SD)	21.6 (3.36)	20.03 (3.73)	t(36) = −0.886	0.381

^†^No female TD controls with a PP peak. Intelligence levels and ages are expressed as mean (SD)

*TD* typically developing, *RPM* Raven progressive matrices, *GIQ* global IQ from Wechsler’s Intelligence Scales, *SD* standard deviation

** *p* < 0.001; * *p* < 0.01

#### Predisposing Factors

Between-group analyses showed that individuals with SIS tended to be older and had higher intelligence levels, measured by either Wechsler’s GIQ or RPM (Table 3a), than those without SIS. The sex ratio was similar between groups containing individuals with or without SIS. The logistic regression model including sex, intelligence, and age as independent variables and SIS as the dependent variable confirmed this result showing that age and intelligence, but not sex, were associated with SIS (Table 4a). We divided the groups according to age and found that an SIS was present in 27.8 % of children between the ages of 2 and 5, 72.2 % of children between the ages of 6 and 13, and 78.4 % of adults and adolescents aged 14 years or more.

**Table 4:** Predisposing factors to a. Special isolated skills and b. Perceptual peaks: age, intelligence (FSIQ or RPM), sex and group

Independent variables	Special isolated skills				
	Coefficient (B)	SE	Wald	*p* value	Exp(B) (odds-ratio)
a.					
Age in years	0.084	0.033	6.402	0.011	1.087
Wechsler’s GIQ z-score	0.391	0.146	7.147	0.008*	1.479
Sex	−0.539	0.518	0.860	0.354	0.584
Constant	0.986	0.707	1.947	0.163	2.680
Age in years	0.101	0.038	7.009	0.008*	1.106
RPMz-score	0.525	0.188	7.808	0.005*	1.690
Sex	−0.606	0.648	0.874	0.350	0.546
Constant	0.406	0.770	0.278	0.598	1.501

Independent variables	Perceptual peaks				
	Coefficient (B)	SE	Wald	*p* value	Exp(B) (odds-ratio)
b.					
Age in years	0.040	0.060	0.441	0.507	1.041
Wechsler’s GIQ z-score	−1.204	0.383	9.882	0.002*	0.300
Sex	1.601	0.906	3.125	0.077	4.958
Group	1.729	0.632	7.481	0.006*	5.636
Constant	−7.205	2.595	7.711	0.005*	0.001
Age in years	−0.020	0.053	0.140	0.708	0.980
RPM z-score	0.137	0.346	0.156	0.693	1.146
Sex	0.924	0.785	1.385	0.239	2.520
Group	2.231	0.596	14.006	<0.001**	9.307
Constant	−5.541	2.185	6.433	0.011	0.004

*GIQ* global IQ from Wechsler’s Intelligence Scales, *RPM* Raven progressive matrices

** *p* < 0.001; * *p* < 0.01

### Study 2: Perceptual Peaks (PP) and Co-occurrence Between Modalities and Types of SIS

#### Preliminary Analyses on Standardized Performance Scores

**Fig. 3:**
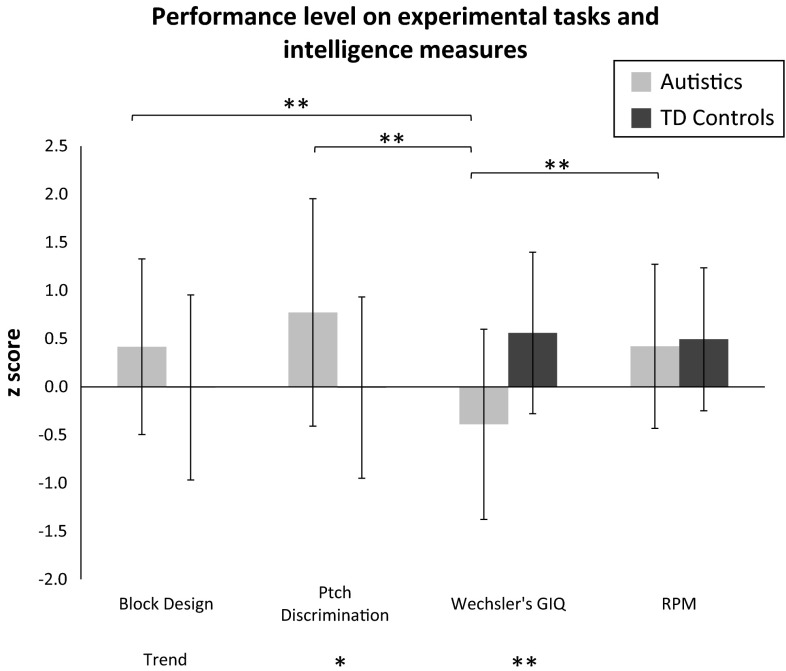
Performance level on experimental tasks (Block design, Pitch discrimination) and on intelligence measures (Wechsler’s Global IQ (GIQ), Raven Progressive Matrices (RPM)). Performance levels are shown in z score for autistics (*light grey*) and the typically developing (TD) controls (*dark grey*). The stars above the brackets, at the top of the graph, represent significance levels for differences in task performance separately for each group. The last line at the bottom of the graph indicates significance levels for between group differences in performance separately for each task and measure. ** *p* < 0.001; * *p* < 0.001

We examined overall group differences in auditory and visual performance for autistic and non-autistic individuals of comparable age and RPM measured intelligence. The autistic group was more sensitive to pitch change than the TD control group (t(69) = −3.077, *p* = 0.003, d = −0.73) and tended to perform better in the BD task, although this latter difference was not significant (trend, t(71) = −1.924, *p* = 0.058, d = −0.45) (Fig. 3). Analysis of between-task differences revealed that the performance of autistic individuals in perceptual tasks was better than their general intellectual functioning when intelligence was measured by Wechsler’s GIQ, but not by RPM. This supports the findings of Dawson et al., which suggest that the intelligence level of autistic individuals is probably underestimated when measured with Wechsler’s GIQ and not RPM (Dawson et al. 2007). There were no statistically significant differences in performance between auditory and visual tasks in TD controls (*p* > 0.01).

#### Prevalence

**Fig. 4:**
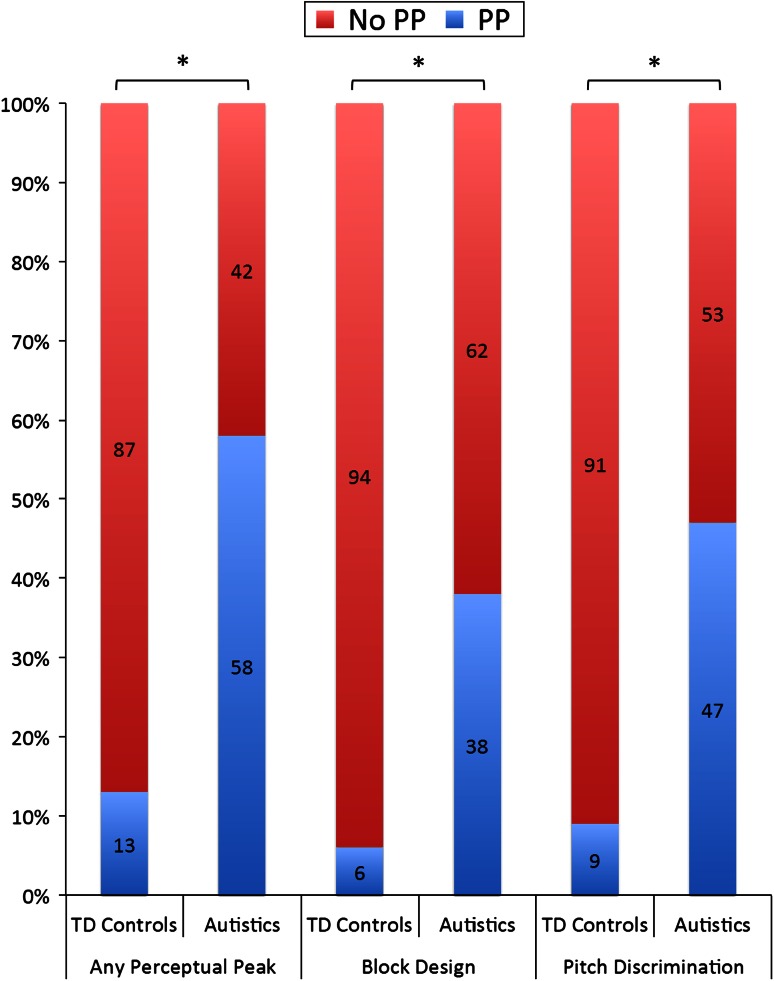
Graph showing the percentage of the sample from Study 2 with (*blue*) and without (*red*) strengths, or “Perceptual Peaks” (PP), in any task and in each task separately. The graph shows the percentages separately for typically developing (TD) controls (*left*) and autistics (*right*). The proportion of individuals with and without PP is significantly different between TD controls and autistics. * *p* < 0.001

Table 3b shows the prevalence of PP (defined as a performance level at least 1 standard deviation above the general intellectual functioning of the particular individual) and the age and intelligence level of participants with or without PP. Figure 4 illustrates the prevalence of PP by modality. The proportion of autistic individuals with PP in at least one modality was 57.5 % (23/40), whereas this figure was only 13.2 % (5/38) for controls. Analysis of participants who successfully completed both empirical tasks (n = 33 per group) showed that auditory and visual peaks were equivalently distributed in autistic (McNemar test *p* = 0.774) and non-autistic individuals (McNemar test *p* = 1.000) (Fig. 4). Logistic regression analysis showed that the chances of having a PP in either task were significantly greater for autistic individuals than for controls (Table 4b; Fig. 4).

Comparison of the prevalence of PP and SIS among autistic individuals showed that the prevalence of PP (57.5 %) was slightly lower than that of SIS found in the unselected large sample of Study 1 (62.6 %). Furthermore, the prevalence of PP (57.5 %) was significantly inferior to that of SIS (88 %) within the subgroup of autistic adolescents and adults with average RPM intelligence (≥25th percentile) and with valid data for both PP and SIS (n = 40) (McNemar test *p* = 0.012).

#### Predisposing Factors

RPM was not associated with PP in either group, whereas Wechsler’s GIQ was associated with PP in autistic individuals only, with a low GIQ favoring the presence of PP. Sex and age were not significantly associated with PP (Table 3b). Logistic regression analysis including sex, intelligence, and age as independent variables and PP as the dependent variable confirmed these results (Table 4b): Wechsler’s GIQ, but not age and sex, showed an independent association with PP across groups.

#### Co-occurrence of Outstanding Abilities in Autistic Individuals

In autistic individuals, the presence of a PP in the pitch discrimination task was not significantly associated with the presence of a PP in the BD task (*p* = 0.140). PP co-occurred in perceptual and auditory tasks in 24 % (8/33) of autistic participants, which slightly exceeds the proportion expected by chance (18 %). None of the control participants had PP in both tasks, which is consistent with the expected co-occurrence of PP by chance (0.55 %). Given the small sample sizes and low probability of the co-occurrence of PP, logistic regression analysis between PPs could not be reliably conducted for controls and the findings of this analysis should be intepreted with caution for autistic individuals.

We found that 86 % of autistic individuals included in Study 2 had at least one SIS. Given the high prevalence of SIS in this subgroup, it was not surprising to find that 83 % (19/23) of individuals who presented a PP (in the BD and/or pitch discrimination task) also presented at least one SIS. We carried out a logistic regression analysis with Wechsler’s GIQ and SIS in music as independent variables and PP in pitch discrimination as the dependent variable. This analysis showed that autistic individuals with an SIS in music were not more likely to have a peak in pitch discrimination than autistic individuals of similar intelligence without an SIS in music (B = 2.989, SE = 1.604, Wald = 3.473, *p* = 0.062, OR 19.858). Similarly, autistic individuals with an SIS in visuospatial activities (e.g. puzzles) or drawing were not more likely to have a PP in the BD task than autistic individuals of similar intelligence without an SIS in visuospatial activities or drawing (*p* = 0.288 and *p* = 0.780, respectively). Furthermore, an SIS in memory was not significantly associated with a PP in the BD task (*p* = 0.953), but an SIS in memory tended to be associated with a peak in pitch discrimination (B = 3.392, SE = 1.504, Wald = 5.089, *p* = 0.024, OR 29.723), when intelligence was controlled for by Wechsler’s GIQ.

## Discussion

### Prevalence of Outstanding Abilities in Autistic Individuals

In this study, we report that the prevalence of outstanding skills (defined as a discrepancy between baseline function and at least one competence) in autistic individuals is two to three times higher than commonly reported in the literature. This is true of strengths defined clinically by standardized parental interviews (SIS) or by laboratory measures (PP). The prevalence of SIS and PP combined was 88.4 % (38/43) in autistic individuals with average intelligence measured by RPM.

We assessed the prevalence of SIS with parental interviews and found that it was 62.6 % in a large group of strictly defined autistic individuals of a wide range of intelligence levels. We defined an SIS as a performance above the individual’s general level of cognitive functioning (relative peak) and above that of others in the general population of comparable age (absolute peak), either at the time of the interview (“current”) or at a previous point in life (“ever”). Differences in methodology may explain the discrepancy between our findings and those reported previously. For example, some studies examined only “current” SIS and excluded SIS that were present at a previous point in life (Bolte and Poustka 2004; Howlin et al. 2009). This may explain why the prevalence reported in these studies is lower than that reported here, because some SIS may disappear with age. Indeed, SIS may not be encouraged, or the discrepancy between autistic and non-autistic performance may only be present at a particular age, as is the case for hyperlexia. Alternatively, improvements in adaptive abilities may accompany loss of skills involving hyperfocus, as autistic people learn and adapt. Another possible source of discrepancy between findings is differences in the populations studied. In our population, the average GIQ on Wechsler’s scale was 87 among the 171 participants (out of 254) who took the test. The population studied by Howlin et al., for instance, was based on a cohort diagnosed in the 1950s to 1980s; therefore, this cohort was probably composed of people with more atypical phenotypes than those included in studies of autism today (Howlin et al. 2009). In addition, only 87/137 participants in this study completed the Wechsler scale, with an overall group IQ estimated at 78. The present cohort can be considered a representative sample of individuals diagnosed according to the current definition of autism. The proportion of individuals with intellectual disability in our cohort: (IQ < 70: M 18 %, F 17 %) is slightly lower than that among autistic individuals in the US population (IQ < 70: M 35 %, F 45 %) (CDC 2008). This difference is partly explained by our decision to exclude ten subjects from our study with an associated medical condition, the majority of whom presented low IQs, and partly by a recruitment bias toward individuals with verbal competence.

We used experimental tasks to investigate PP, which we defined as a standardized performance level at least 1 standard deviation above the subjects own level of cognitive functioning (relative peak), as defined by Wechsler’s full scale IQ. PPs were identified in 57.5 % of autistic individuals: 38 % of autistic individuals presented a relative strength in the BD test and 47 % displayed a relative strength in the pitch discrimination test (compared to 6 and 9 % in TD individuals of comparable age and intelligence measured by RPM, respectively). This is a conservative estimate of prevalence, because participants with formal music/drawing experience were excluded to avoid bias from the effect of training. The BD task completed by participants in our study was more difficult than that used by Howlin et al. and it was designed to be optimally solved through a local processing approach with more autonomy of configural processing systems. Nonetheless, the prevalence of the BD peak reported here is close to that reported by Howlin et al. (26.4 %) with the classical BD task and that reported by Caron et al. (2006) (47 %), who used a more inclusive relative peak definition than the “relative + absolute” peak definition used by Howlin’s team. The high prevalence of PP in our study may be related to our inclusion of a pitch discrimination task. Superior pitch discrimination is arguably the most replicated peak of ability in autism (Mottron et al. 2013a). Significant differences in pitch discrimination can be found in groups with as few as 12 autistic participants (Bonnel et al. 2010, 2003; Jones et al. 2009). Therefore, a large proportion of PP in autistic individuals involve superior pitch discrimination.

### Co-occurrence of Autistic Outstanding Abilities

The co-occurrence of perceptual strengths in auditory and visual modalities, including PP in the pitch discrimination and the BD task, was rather low and occurred in only eight (24 %) autistic subjects and none of the controls. This finding contrasts with those of a prior study showing a link between pitch labeling and both memory and BD scores (Heaton et al. 1998). It is therefore possible that the completion of BD tasks may be more closely related to pitch labeling abilities than to pitch discrimination abilities. Another difference between our study and that of Heaton et al. (1998) is the age of participants (20.8 vs. 9.9 years old, respectively). The association between perceptual abilities may be strong in young individuals and may decrease with age.

We also examined the co-occurrence of talents and strengths in the same individual. More than 71.7 % of autistic individuals with an SIS had two or more SIS, which is higher than previous findings of 21 % (5/24) reported by Howlin et al. 2009. Given this high prevalence of SIS in autistic individuals, it was not surprising to find that 83 % (19/23) of autistic individuals who presented a PP also presented at least one SIS. Therefore, the strong relationship between the most frequent SIS (memory, found in 52.5 % of autistic individuals with an SIS) and PP (pitch discrimination, found in 77.2 % of autistic individuals with a PP) may simply be due to their combined high prevalence. This, along with the inclusion of pitch discrimination in addition to BD as a strength in the current study may account for differences between our findings and those of Howlin et al., who reported that cognitive peaks and SIS overlap in only a small proportion of autistic individuals (8.6 %).

### Predisposing Factors of Autistic Outstanding Abilities

#### Intelligence

Another main finding of our study is that the prevalence of SIS and PP is related to cognitive functioning, although this relationship differs according to SIS and PP and to the method used to measure intelligence. Individuals with SIS tended to have higher intelligence levels than those without SIS, as measured by either Wechsler’s IQ or RPM, consistent with previous findings (Happe and Vital 2009; Howlin et al. 2009; Rapin 1996; Vital et al. 2009). In addition, no individual judged as having at least one talent (SIS) presented a non-verbal IQ below 50 (Howlin et al. 2009). By contrast, a lower Wechsler-defined IQ favored the presence of perceptual strengths across domains, which is consistent with our definition of relative strengths as a discrepancy between verbally mediated Wechsler-defined general intelligence and performance in perceptual tasks. Individuals with a low IQ were more likely to have a perceptual strength in pitch discrimination than individuals with a moderate or high IQ (B = −2.109, SE = 0.644, Wald = 10.727, *p* = 0.001). The same was true, to a lesser extent, in the block task (B = −0.930, SE = 0.419, Wald = 4.920, *p* = 0.027). However, this relationship may not be true of IQs under 50 (Miller 1999). RPM was not related to the presence of PP in either task, suggesting that outstanding performance in these activities may be related to a factor other than general intelligence.

One way to account for these findings is to distinguish “true” and “false” intellectual disability in autism. Autistic individuals most frequently display strengths in perceptual or non-verbal tasks, such as the BD test, whereas baseline intelligence is frequently measured by verbal tasks. As a result, the fluid intelligence of autistic individuals can only be accurately determined with strictly non-verbal tasks (such as the BD task) or a non-verbal problem solving test, such as RPM (Barbeau et al. 2013; Dawson et al. 2007). A “true” intellectual disability is revealed by poor performance in these nonverbal tasks. By contrast, a “false” intellectual disability may be defined as an impaired verbal IQ in autistic individuals with limited speech abilities, revealed by a non-verbal peak (BD or RPM) in presence of a low verbal baseline.

#### Age

The prevalence of SIS among autistic individuals increased with age and was 27.8 % between the ages of 2 and 5, 72.2 % in school age children and 78.4 % in adolescents and adults. This observation suggests a bias in the reporting of extremely young talents, around and before 5 years of age (Aram and Healy 1988; Horwitz et al. 1965; Miller 1989; Selfe 1983). Nonetheless, some talents emerge between 8 and 15 years (Dubischar-Krivec et al. 2009; Soulieres et al. 2010) and verbal and non-verbal skills become increasingly dissociated over time (Joseph et al. 2002). Experience is expected to play a role in autistic people, as it does in non-autistic individuals. By contrast, the prevalence of PP was not associated with age and experience, as reported by previous groups (Heaton et al. 2008b; Mottron et al. 2013a), indicating that PP are at least partly based on early, genetically determined alterations of the perceptual brain architecture.

#### Sex

The presence of SIS and PP was similar between male and females in our group, but the sample size of the female group limits the strength of this conclusion. In the study by Howlin et al., there were many more males than females presenting savant skills (32 M:7F), but this ratio was similar to the sex ratio (M:F) of the total group (Howlin et al. 2009). In another study exploring the prevalence of savant syndrome in several conditions including autism, Treffert reported that males outnumbered females by a ratio of approximately 6:1, which is higher than the sex ratio of 4:1, which is typically reported for autistic disorders (Treffert 2009). We found that the sex ratio of savant skills in autism is similar to the sex ratio of individuals with the condition, which is consistent with the findings of Howlin et al. By contrast, Vital et al. studied on a group of individuals with autistic traits (but not necessarily diagnosed with autism) and found sex differences in the prevalence of savant skills. However, this finding was significant only in the univariate analysis and had a very small effects (Vital et al. 2009). Overall, these studies suggest that sex itself may not be a primary contributing factor to the development of outstanding abilities; however, studies including a larger number of females are needed because this is a recurring limitation among studies, including ours.

### Contribution to the Understanding of Strengths and Talents

Many autistic people possess several talents and/or strengths; however, possession of a strength in one modality does not increase the chances of having a talent in the same modality. Therefore, the development of clinically defined SIS in a particular modality does not appear to be directly related to the level of functioning of perceptual processes of the same modality, as assessed by the empirical measures used in this study. We recently examined performance in tasks investigating low and mid-level auditory and visual processing in the same participants used in the current study (Meilleur et al. 2014). This analysis revealed the existence of plurimodal covariation between tasks that was independent of general intelligence and specific to the autistic group, indicating that a common underlying “p” factor drives perceptual abilities differently in autistic and non-autistic individuals. Overall, these findings suggest that exceptionality (strengths or talents) and perceptual performance in autism are the result of largely independent mechanisms. Perceptual encoding across modalities is altered in autistic individuals, and this is probably mediated by a factor other than intelligence. This alteration may be genetic in nature, and related to the over-functioning of mechanisms of synaptic plasticity (see Mottron et al. 2014 for a review and model). This alteration is beneficial to certain, but not all, low and mid-level level operations in both modalities, including pitch (Heaton et al. 2008a), luminance (Bertone et al. 2005), spatial frequencies (Bennett and Heaton 2012), auditory local processing (Bouvet et al. 2014b), and visual search (Plaisted et al. 1999). Thus, perception is modified in its underpinnings, with potential positive or negative consequences. Perceptual alterations directly resulting from causal mutations, experience and differences in the overall genetic background, may determine the development of talents in a particular subgroup of individuals. The dependence of these interactions on an “exposure” and “material availability” component for domain specific talents may be responsible for the relatively low overlap between talents and strengths in the same modality.

### Strengths and Limitations

The strengths of this study include its relatively large sample size of autistic individuals diagnosed according to standardized criteria, the combined use of clinical and experimental studies and the exclusion of neurodevelopmental conditions. We also used well-defined criteria to investigate savant skills and PP and collected data according to standardized methods. However, certain limitations restrict the generalization of our findings. We included only autistic individuals diagnosed according to DSM-IV criteria and we included no individuals with Asperger’s syndrome. The population under study had a slightly higher average IQ than other large populations of autistic individuals. Finally, ADI-R may be affected by a positive bias from the parents and only provides a single question per domain, but this may have been compensated by the fact that several domains of SIS were investigated.

## Conclusion

A discrepancy between baseline functioning and at least one competence is very common in autism. The development of such “Special Isolated Skills” is correlated with age and intelligence, but the occurrence of PP and the presence of other “Special Isolated Skills” in different perceptual modalities appears to be relatively independent. These observations suggest that experience is the main factor involved in the development of such strengths and/or talents and that genetically defined modifications affect perceptual encoding.

## Electronic supplementary material

Below is the link to the electronic supplementary material.

Supplementary material 1 (DOC 35 kb)

## Footnotes

1
homepages.abdn.ac.uk/j.crawford/pages/dept/SingleCaseMethodology.htm.
