# Supplementary material for: Prevalence of Clinically and Empirically Defined Talents and Strengths in Autism
Source: J Autism Dev Disord. 2014 Nov 6;45(5):1354–67. doi: 10.1007/s10803-014-2296-2 (PMC4544492; doi:10.1007/s10803-014-2296-2)
Supplement: Supplementary file 1 — Supplementary material 1 (DOC 35 kb) [file 10803_2014_2296_MOESM1_ESM.doc]

| **Table S1.** Prevalence of SIS “Current” compared to “Ever” in full sample and separated by age groups | | | | | |
| --- | --- | --- | --- | --- | --- |
|  |  |  | SIS % | | McNemar |
|  | n | Age mean (SD); range | Current | Ever | p value |
| **Full Sample** | 254 | 11.35(7.998); 2-39 | 59.4 | 62.6 | .008 |
| Preschool | 65 | 3.46(.937); 2-5 | 28 | 28 | 1.000 |
| School Age | 115 | 9.33(2.097); 6-13 | 69 | 72 | 0.125 |
| Adolescents/Adults | 74 | 21.41(7.052); 14-39 | 73 | 78 | 0.125 |

| **Table S2.** Percentage (n loss/n total) of subject with loss of skill in the full sample and by age groups | | | | | | | |
| --- | --- | --- | --- | --- | --- | --- | --- |
|  | Memory | Visuospatial | Reading | Drawing | Music | Computation | Any SIS |
| **Full Sample** | 4.7(6/127) | 22.1 (17/77) | 16.3(8/49) | 15.8(6/32) | 10.3 (4/39) | 16.7(6/36) | 5.0 (8/159) |
| Preschool | 0(0/12) | 28.6 (2/7) | 0 (0/6) | 0 (0/1) | 0(0/9) | 0 (0/3) | 0 (0/18) |
| School Age | 4.3(3/69) | 15.8 (6/38) | 11.1 (3/27) | 13.3 (2/15) | 5.6(1/18) | 16.7 (3/18) | 4.8 (4/83) |
| Adolescents/Adults | 6.5(3/46) | 28.1 (9/32) | 31.3 (5/16) | 13.6 (3/22) | 25(3/12) | 20 (3/15) | 6.9 (4/58) |
